# Development of a Triage-Level Predictive Model for Hospitalization in the Emergency Department

**DOI:** 10.3390/jcm15051901

**Published:** 2026-03-02

**Authors:** Daniel Trotzky, Yoav Preisler, Almog Amoyal, Gal Pachys, Jonathan Mosery, Aya Cohen, Shiran Avisar, Tomer Ziv Baran

**Affiliations:** 1 Medical Management, Tel Aviv Sourasky Univeristy Medical Center, Affiliated to the Gray Faculty of Medical & Health Sciences, Tel Aviv University, Tel Aviv 6423906, Israel; 2 Department of Epidemiology and Preventive Medicine, School of Public Health, Gray Faculty of Medical & Health Sciences, Tel Aviv University, Tel Aviv 6139001, Israel; 3 Department of Internal Medicine “T”, Tel Aviv Sourasky University Medical Center, Affiliated to the Gray Faculty of Medical & Health Sciences, Tel Aviv University, Tel Aviv 6423906, Israel; 4 Gray School of Medicine, Gray Faculty of Medical & Health Sciences, Tel Aviv University, Tel Aviv 6139001, Israel; 5 Department of Emergency Medicine, Shaare Zedek Medical Center, Jerusalem 9103102, Israel; 6 The School of Medicine, The Hebrew University, Jerusalem 9190401, Israel; 7 Department of Emergency Medicine, Shamir Medical Center (Formerly Assaf-Harofeh), Zerifin 7030000, Israel; 8 Division of Emergency Medicine & Emergency Preparedness, Tel Aviv Sourasky University Medical Center, Affiliated to the Gray Faculty of Medical & Health Sciences, Tel Aviv University, Tel Aviv 6423906, Israel

**Keywords:** emergency department, triage, crowding, hospitalization, prediction model

## Abstract

**Background/Objectives**: Overcrowding in the emergency department (ED) is a global health issue. Early prediction of expected hospitalizations, based on parameters available from triage, is essential to enhance patient transfer from the ED to departments, thereby reducing ED congestion. **Methods**: A historical cohort study included patients who visited two tertiary referral medical centers located in the center of Israel. Data derived from one medical center (MC-A) was used to build the prediction model and to test it, and data from the second medical center (MC-B) was used to validate it. Variables collected included age, sex, triage level, vital signs, initial admitting diagnosis, medical referrals, mode of arrival, time of arrival according to hospital shifts (morning, evening, and night), weekday (workdays/weekend), season, fall risk assessment, and significant comorbidities. Logistic regression was used to build the model, and the area under the ROC curve (AUC) and the discrimination slope (DS) were used to evaluate it. **Results**: The final cohort included 1436 patients: 1256 patients from MC-A and 180 from MC-B. The patients were divided randomly into a learning group (*n* = 879), a test group (*n* = 377), and a validation group (*n* = 180). We found that higher triage level (urgent+: OR 1.45, *p* = 0.039), lower O_2_ saturation (<95%: OR 3.32, *p* < 0.001), malignancy (OR 1.81, *p* = 0.044), cardiovascular disease (OR 2.93, *p* < 0.001), neurologic illness (OR 2.07, *p* = 0.014), arrival during the weekend (OR 1.57, *p* = 0.014), and fall season (OR 1.81, *p* = 0.003) were associated with higher probability of hospital admission. Our model showed a similar acceptable discrimination ability in all groups (learning: AUC = 0.77, 95%CI 0.73–0.80, and DS = 19%; testing: AUC = 0.76, 95%CI 0.70–0.82, and DS = 17%; validation: AUC = 0.71, 95%CI 0.61–0.80, and DS = 18%). **Conclusions**: The proposed prediction model can be easily implemented in hospital systems to provide management with an expected number of ED patient hospitalizations in the coming hours. The model can enhance patient flow, thereby reducing crowding in the ED.

## 1. Introduction

Emergency department (ED) crowding is a global health issue [1]. Prolonged ED waiting time and extended visit duration reduce the quality of medical care, compromise patient safety, and increase the risk for adverse events [2,3]. Moreover, prolonged waiting time is associated with patient dissatisfaction [4,5,6] and an increased number of patients who left the ED before medical evaluation [7,8]. In 2007, the United States Institute of Medicine considered ED crowding a “national epidemic” [9]. In addition to the compromise of patient privacy and confidentiality, ED crowding and waiting time frequently cause frustration among staff [10].

Several factors affect the flow of patients in the ED, from arrival and in-hospital management to admission or discharge from the hospital [11,12]. An umbrella review conducted by Samadbeik et al. found that most interventions for enhancing patient flow in the ED focused on the ‘internal ED’ phase, including structural reorganization and operational changes, while few others investigated the ‘pre-ED’ phase and ‘post-ED’ phase [13].

Bittencourt et al. suggested that physicians working in triage, either alone or with nurses, may provide accelerated diagnostic procedures and treatments, resulting in reduced length of stay and waiting time per patient [14]. Nevertheless, other studies showed that team triage had no clear benefits and not enough evidence regarding its advantages [15,16].

Several models aimed at predicting hospitalization have been previously proposed. One model focused on a fatality prediction approach [17], while other models used machine learning techniques that were based on laboratory tests and demographic parameters [18,19,20]. Another study examined the utility of a machine learning model to predict hospitalization in COVID-19 patients [21].

The literature is inconsistent regarding the effectiveness of strategies and interventions to reduce ED crowding and waiting time per patient. Many papers that were reviewed were either pilot studies or quality improvement projects that lacked thorough evaluation against comparator groups. Inconsistencies in patients’ assessment and management to improve patient flow emphasize the need to establish measures and evidence-based solutions [22,23].

Therefore, this study aimed to provide a prediction model for hospital admissions within the first few hours of arrival, utilizing parameters from triage, primarily the initial patient evaluation in the ED. Such a model may enhance patient flow from the ED to in-hospital service and reduce ED crowding by providing the department with an early indication of the need for patient hospitalization. In addition, it may improve both the patients’ and the medical teams’ satisfaction.

## 2. Materials and Methods

### 2.1. Setting

A historical cohort study was conducted at the Tel Aviv Sourasky Medical Center (MC-A), a 1200-bed medical center, and the Shamir Medical Center (MC-B), a 900-bed medical center. Both MC-A and MC-B are university-affiliated hospitals located in the center of Israel. MC-A serves an urban population of one million people, and MC-B serves an urban and rural population of one million people. Both medical centers are tertiary referral centers with approximately 230,000 and 155,000 ED visits each year, respectively. MC-A is also a level 1 trauma center. The ED’s operate all year round, around the clock. The ED staff includes attending- and resident-level physicians as well as certified emergency medicine registered nurses and physician assistants (PAs). Data derived from MC-A was used to build the prediction model, and data from MC-B was used to validate it. This study was approved by the local ethics committee of both medical centers (903-18-TLV, 113-21-ASF).

A random sample of 1300 adult patients (age ≥ 18) who visited the ED of MC-A between January 2018 and December 2018, and a random sample of 180 adult patients (age ≥ 18) who visited the ED of MC-B between September 2023 and August 2024, were included in the study.

The ED operates in three shifts: the morning shift (7:00 a.m. to 3:00 p.m.), the evening shift (3:00 p.m. to 11:00 p.m.), and the night shift (11:00 PM to 7:00 a.m.). In MC-A, at least three patients per day from each shift were randomly selected. In MC-B, 15 patients per month were randomly selected, five from each shift.

The study population was divided into three groups: learning, testing, and validation. The MC-A patients were randomly divided into two groups. The first group included a random sample of seventy percent of the patients and was used as the learning group in order to identify predictors for hospitalization (admission to departments) and to build the prediction model. The second group was the testing group and included the rest of the patients (30%) from MC-A. The third group was used to validate the model (validation group) and included the patients from MC-B.

### 2.2. Data Source and Study Variables

All data were collected from patient triage as documented by the registered nurses in the electronic medical chart. Variables collected included age, sex, triage level, vital signs, initial admitting diagnosis, medical referrals, mode of arrival, time of arrival, weekday (workdays/weekend), season, fall risk assessment, and significant comorbidities.

Initial admitting diagnoses were categorized as medical or surgical/trauma. “Medical” admitting diagnoses included a variety of internal diseases, such as neurologic, cardiovascular, and infectious diseases. Admitting diagnoses categorized as “surgical/trauma” included otolaryngology, surgical, urology, and trauma issues. Triage level in both ED’s uses the Canadian Triage and Acuity Scale (CTAS), which prioritizes patients’ evaluation according to five levels: level 1 (resuscitation), level 2 (emergent), level 3 (urgent), level 4 (less urgent), and level 5 (non-urgent). We divided the triage levels into three categories: immediate/emergency (1–2), urgent (3), and semi- or non-urgent (4–5). Vital signs collected included pulse, blood pressure, O_2_ Saturation, and temperature. The ED arrival time was categorized by hospital shift (morning, evening, and night). Seasons were categorized as autumn, winter, spring, or summer. Fall risk was evaluated by a registered nurse at the triage level. The mode of arrival to the ED was categorized into three options: private vehicle, basic life support ambulance (BLS), and advanced life support ambulance (ALS). Major comorbidities included pre-existing disease of cardiovascular, neurologic, or respiratory origin, and malignancy.

### 2.3. Sample Size

Sample size was calculated using a significance level of 5% and a power of 80%. In Israel, approximately a quarter of those who visit the ED are hospitalized [24]. Therefore, a ratio of 1:3 was used to calculate the required sample size. In order to identify predictors with a small effect (d = 0.2) on hospital admission, 1048 patients were needed. In addition, the same size that was required to describe the area under the receiver operating characteristic curve (AUC ROC) was also calculated. We assumed an AUC ROC of 0.8 with a 95% confidence level width of 0.2. Using these assumptions, 155 patients were needed.

### 2.4. Statistical Analysis

Categorical variables were reported as numbers and percentages. Continuous variable distribution was evaluated using histograms and Q-Q plots. Since not all continuous variables were normally distributed, they were reported as median and interquartile range (IQR). The chi-square test was used to compare categorical variables between those who were admitted and those who were not. The Mann–Whitney test was applied to compare the continuous variables. Variables were categorized using the chi-square automatic interaction detection and Classification and Regression Trees methods. Variables that were found to be associated with hospital admission, i.e., those that were categorized, were included in the multivariable model. Logistic regression using the backward elimination method for variable selection was used for the multivariable analysis. The backward elimination method selection criterion was based on the likelihood ratio test, and *p* > 0.1 was used as the criterion for variable removal. The Nagelkerke R^2^ and the Brier score of the regression were calculated. In addition, a penalized logistic regression model was fitted using the least absolute shrinkage and selection operator (LASSO) technique. This method is particularly advantageous in settings with correlated predictors or when overfitting is a concern. The optimal degree of penalization was determined using 10-fold cross-validation. To improve model stability, 1SE was applied for variable selection for the final model. The models were used to estimate the probability of department admission for each participant in the learning, testing, and validation groups. The area under the receiver operating characteristic (ROC) curve (AUC) and discrimination slope (DS) were used to assess the discrimination ability of the model. Calibration was assessed with calibration plots comparing predicted and observed risks. Clinical usefulness was examined using decision curve analysis (DCA), which estimates net benefit across a range of threshold probabilities relative to “hospitalize all” and “hospitalize none” strategies. A nomogram was developed to enhance the clinical usability of the prediction model. Using the coefficients from the logistic regression, each predictor was assigned a point value proportional to its effect size. The total score corresponds to an estimated probability of the outcome, enabling individualized risk assessment without the need for an online calculator or software. All statistical tests were two-sided, and *p* < 0.05 was considered statistically significant. SPSS (IBM SPSS Statistics for Windows, version 29.0.2, IBM corp., Armonk, NY, USA, 2023) and R (version 4.5.1, R Foundation for Statistical Computing, Vienna, Austria, 2025) were used for all statistical analyses.

## 3. Results

### 3.1. Study Population

The final cohort included a total of 1436 patients: 1256 patients from MC-A and 180 from MC-B. The patients were divided into a learning group (*n* = 879), testing group (*n* = 377), and validation group (*n* = 180). Table 1 summarizes the characteristics of patients among the learning, testing, and validation groups. Overall, the characteristics were similar between groups. However, a higher triage level (urgent+) was more dominant in the validation group. Similarly, cardiovascular, neurological, and respiratory comorbidities and arrival by ALS ambulance were more common in the validation group. The hospital admission rate was similar in the learning, testing, and validation groups (26.3%, 26.3%, and 28.3%, respectively).

### 3.2. Predictors of Hospital Admission

Among the learning group, older age, higher triage level, higher pulse, higher systolic blood pressure, lower O_2_ saturation, arrival by ambulance, arrival on weekends and during autumn, fall risk, and having comorbidities were associated with increased risk of hospital admission (Table 2).

The multivariable analysis, using the backward selection method, showed that higher triage level (urgent+: OR 1.45, *p* = 0.039), lower O_2_ saturation (<95%: OR 3.32, *p* < 0.001), malignancy (OR 1.81, *p* = 0.044), cardiovascular disease (OR 2.93, *p* < 0.001), neurologic illness (OR 2.07, *p* = 0.014), and arrival during the weekend (OR 1.57, *p* = 0.014) and in autumn (OR 1.81, *p* = 0.003) are associated with a higher probability of hospital admission. Fall risk tends to be associated with hospital admission (OR 1.48, *p* = 0.053) and is also included in the final model. Table 3 summarizes the odds ratios and the model coefficients.

According to the multivariable model, the probability of patients being admitted to a department is defined by the following equation:

p (admission) = 1/(1 + e^(−z))

where p (admission) is the probability of being admitted, e is equal to 2.718, and z = −2.248 + 0.369 (if triage level ≥ urgent) + 1.199 (if O_2_ saturation <95%) + 0.393 (if fall risk assessment) + 0.592 (if malignancy presents) + 1.073 (if cardiovascular illness presents) + 0.727 (if neurologic illness presents) + 0.453 (if arrival upon weekend period) + 0.594 (if arrival upon autumn season).

A second multivariable analysis, using a penalized regression for variable selection, revealed the same list of predictors. The probability of patients being admitted to a department, as estimated by the penalized regression model, is described in Appendix A.

### 3.3. Discrimination Ability and Model Calibration

**Figure 1:**
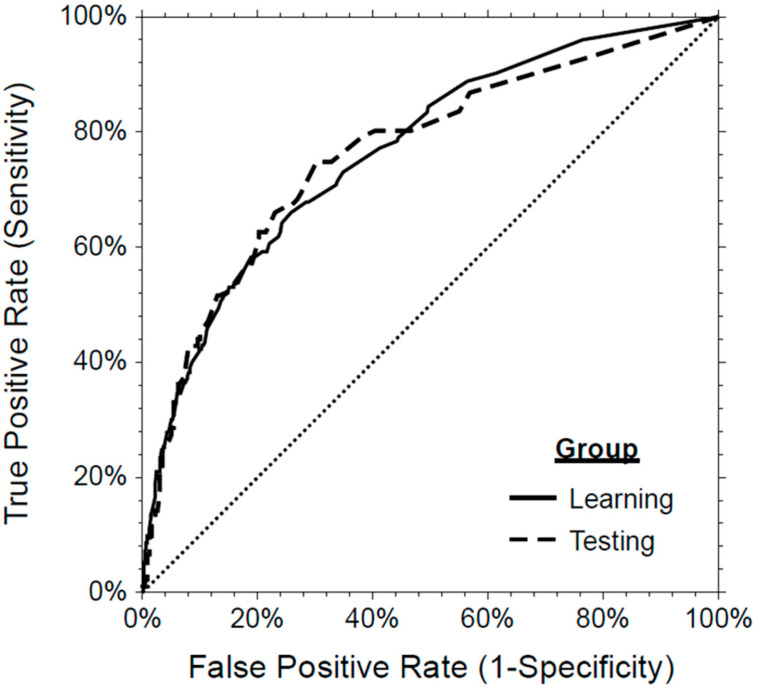
Receiver operating characteristic (ROC) curve demonstrating the discrimination ability of the model to predict hospitalization.

**Figure 2:**
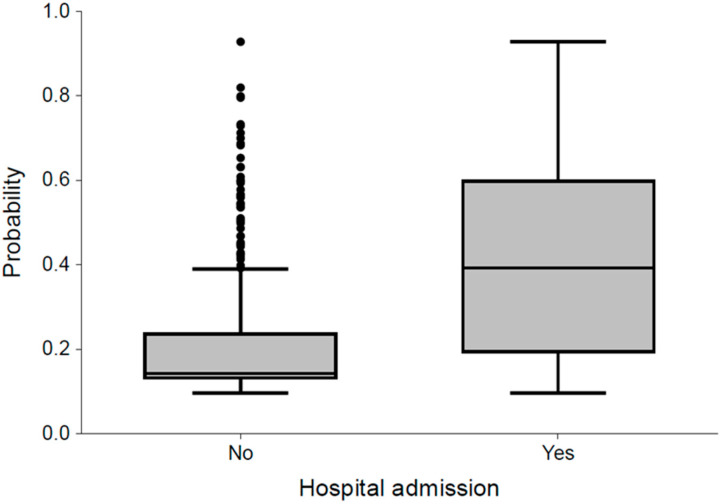
Box and whisker plot of the predicted probability of hospitalization.

To evaluate the discrimination ability and the goodness-of-fit of the model, the area under the ROC curve, the discrimination slope (DS), pseudo R^2^, Brier score, and the Hosmer–Lemeshow test were calculated. For the logistic regression using the backward selection method, the pseudo R^2^ (Nagelkerke R^2^) of the model was 0.247, and the Brier score was 0.158. The Hosmer–Lemeshow test was not significant (*p* = 0.572), which indicates that the model fits the data. In addition, the model showed an acceptable discrimination ability in groups (learning: AUC = 0.77, 95%CI 0.73–0.80, and DS = 19%; testing: AUC = 0.76, 95%CI 0.70–0.82, and DS = 17%; validation: AUC = 0.71, 95%CI 0.61–0.80, and DS = 18%). A ROC curve and a box and whisker plot demonstrate the discrimination ability of the model as presented in Figure 1 and Figure 2.

The penalized logistic regression model showed acceptable discrimination ability in each group (learning: AUC = 0.76, 95%CI 0.73–0.80, and DS = 14%; testing: AUC = 0.77, 95%CI 0.71–0.83, and DS = 13%; validation: AUC = 0.72, 95%CI 0.63–0.82, and DS = 14%), with similar AUCs and lower DSs compared to those of the logistic regression using the backward selection method.

**Figure 3:**
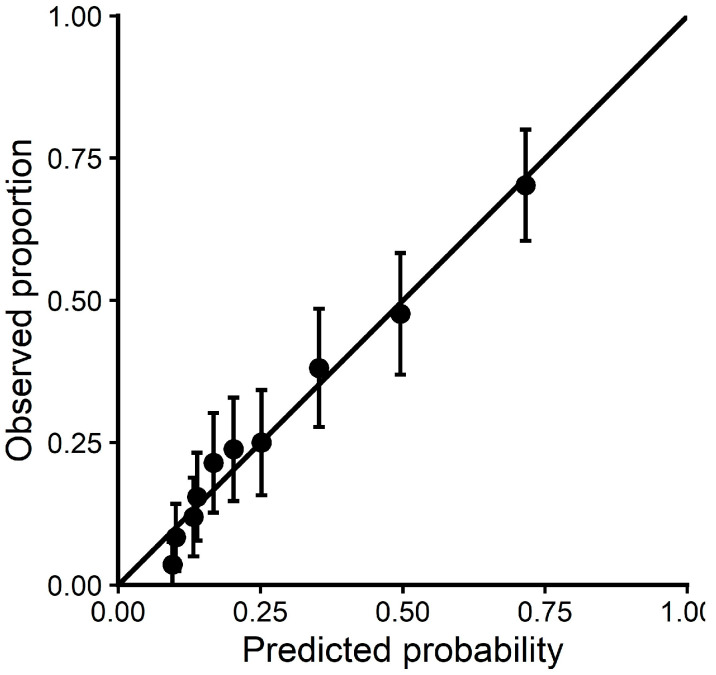
Calibration plot showing the observed vs. predicted event rates (decile-based calibration).

Since the logistic regression using backward selection is more intuitive and yielded similar AUCs, the calibration plot was drawn only for this regression (Figure 3). Overall, as shown in the plot, the model had good calibration, and with only a small overestimation at the lower risk of hospital admission. However, since lower probabilities are less important for the purpose of the current study, no adjustments were applied.

### 3.4. Decision Curve Analysis

**Figure 4:**
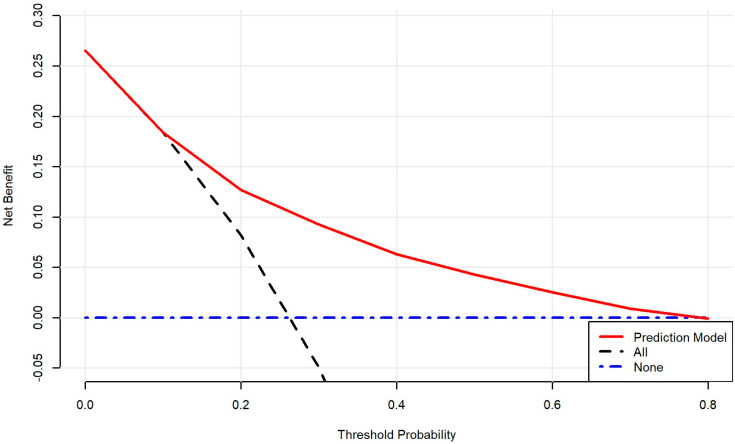
Decision curve analysis demonstrating the net benefit across a range of threshold probabilities.

To estimate the clinical utility of the logistic regression using backward selection, a decision curve analysis was performed. The decision curve analysis demonstrated in Figure 4 shows that the prediction model provided a consistently higher net benefit across a wide range of clinically relevant threshold probabilities compared with the default strategies of hospitalizing all patients or none. The model’s net benefit curve remained above both reference strategies throughout most of the threshold range, indicating that using the model to guide decision-making would result in a more favorable balance between true-positive and false-positive classifications. These findings suggest that the model offers meaningful clinical utility and improves decision quality relative to non-model-based approaches.

### 3.5. Simple Prediction Tool

**Figure 5:**
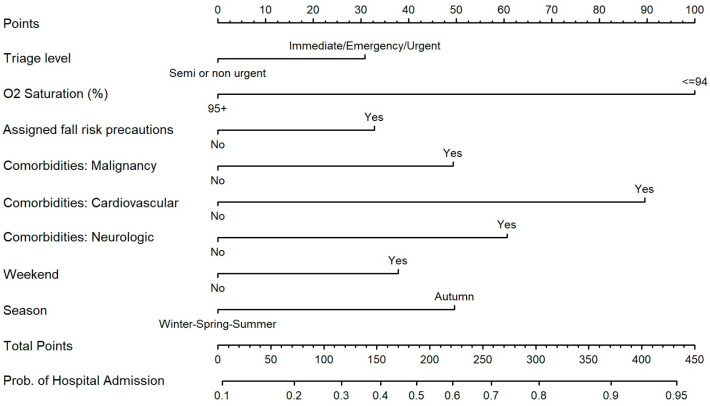
Nomogram for predicting probability of hospital admission.

A nomogram based on the logistic regression model using backward selection was built to enable a simple-to-use and intuitive predictio tool. The nomogram translated the logistic regression model into a point-based tool for individualized prediction of hospital admission. Each predictor contributed a specific number of points proportional to its relative weight in the model. Low oxygen saturation (≤94%) was the strongest contributor, adding 100 points. Cardiovascular comorbidity contributed 90 points, neurologic comorbidity contributed 61 points, and malignancy contributed 49 points. Additional contextual factors also influenced the total score: autumn season added 50 points, weekend presentation added 38 points, and assignment of fall risk precautions contributed 33 points. Summing up these values yields a total point score that corresponds to an estimated probability of hospital admission on the nomogram’s risk scale. The conversion of the total point to hospitalization probability is also available in Appendix A. This structure allows clinicians to visually integrate multiple predictors and derive an individualized risk estimate in a straightforward and interpretable manner (Figure 5).

## 4. Discussion

Crowding in the ED is a widespread global phenomenon with acute adverse effects on the entire hospital. It has a negative impact on the quality of care, and strains have already limited hospital resources [25,26]. Overcrowding decreases the ability of the medical team to provide critical services on time to patients suffering from medical emergencies [27,28]. Previous papers suggested many strategies to mitigate this issue [29]; however, they have had little impact on ED crowding and patient flow, management, and safety [30]. A previous retrospective study of patients who visited the ED in Singapore demonstrated a prediction model for hospitalization that included demographic, administrative, and clinical data routinely collected at triage. However, this model did not include vital signs, chief complaints, the existence of a medical referral, and type of injury. Moreover, it was a single-center study [31]. Another single-center study extracted data including demographic, clinical, socio-economic, triage, vital signs, pathology information, and keywords in electronic notes to predict hospital admission [32].

Previous studies suggested that a clinician performing triage (either a physician or another trained medical caregiver) may reduce ED crowding and length of stay [33,34,35]. However, evidence regarding the effectiveness of this intervention is inconclusive due to the heterogeneity of providers’ programs, and local and site-specific factors [36,37,38]. Additionally, performance of laboratory blood tests and imaging studies in the ED was shown to affect the length of stay [39,40]. In contrast, the current work focused on established parameters and patient data available at triage, without the need for physician assessment or more resource-intensive assessments, including laboratory and imaging analysis.

The multivariable analysis demonstrated that urgent (and greater) triage level, de-saturation (<95%), and cardiovascular, neurologic, and malignancy comorbidities were all associated with increased risk of admission. Patient arrival during the weekend and autumn, as well as the existence of fall risk, were also correlated with a higher probability of hospital admission.

The study has several strengths. Data was collected from a large cohort of patients. Furthermore, as shown above, our model had an acceptable discrimination ability. Data were derived from the electronic medical records (EMR) of two high-volume hospitals that serve both urban and rural populations. The study design included three groups: learning, testing, and validation, which strengthens statistical accuracy. The major strength of the study is its capacity to use readily available and basic patient information collected at triage to provide departments with early notification of the likelihood of patient hospitalization. Notably, our model does not necessitate physician evaluation, blood tests, or imaging modalities to make this assessment.

## 5. Limitations

Our study has a few limitations. First, due to its retrospective nature, some variables and data regarding patients’ characteristics might lack accuracy. However, it represents the real-world complex nature of ED activity. Second, the two medical centers are high-volume hospitals with a wide range of patients visiting the ED each year. The inclusion of two centers during different timeframes introduces potential temporal and site-related bias. Data and conclusions derived from these ED’s might not be applicable to all community hospitals around the world. Each hospital might have a different cutoff for hospitalization due to physician assessment, patients’ preferences, crowding of the hospitalization wards, and the level of support and treatment options in out-of-hospital care. Thus, we do not offer a cutoff point for hospital admission, and every hospital should define its own threshold for admission.

## 6. Conclusions

We developed a simple-to-use prediction model for hospitalization that utilizes information readily available at triage that may enhance patient flow, thereby reducing crowding and frustration from ED congestion and long wait periods.

## Figures and Tables

**Table 1:** Characteristics of patients among the learning, testing, and validation groups.

	Group		
	Learning	Testing	Validation
	(*n* = 879)	(*n* = 377)	(*n* = 180)
Demography			
Age (years), median (IQR)	50 (31–71)	47 (33–69)	50 (29–70)
Female, *n* (%)	434 (49.4%)	193 (51.2%)	94 (52.2%)
Urgency			
Triage levels, *n* (%)			
Immediate/emergency (1–2)	60 (6.8%)	22 (5.8%)	15 (8.3%)
Urgent (3)	371 (42.2%)	164 (43.5%)	124 (68.9%)
Semi- or non-urgent (4–5)	448 (51.0%)	191 (50.7%)	41 (22.8%)
Pulse (bpm), median (IQR)	80 (70–91)	83 (71–94)	80 (71–92)
Blood pressure (mmHg), median (IQR)			
Systolic	136 (124–153)	136 (122–154)	129 (115–148)
Diastolic	79 (69–88)	78 (70–88)	78 (71–85)
O_2_ Saturation (%), median (IQR)	98 (97–100)	98 (97–100)	98 (97–100)
Temperature (°C), median (IQR)	36.8 (36.6–37.0)	36.7 (36.6–37.0)	36.7 (36.6–37.0)
Entry information			
Initial admitting diagnosis, *n* (%)			
Medical	580 (66.0%)	257 (68.2%)	114 (63.3%)
Surgical/trauma	299 (34.0%)	120 (31.8%)	66 (36.7%)
Medical referral, *n* (%)	371 (42.2%)	174 (46.2%)	87 (48.3%)
Mode of transportation, *n* (%)			
Private vehicle	617 (70.2%)	262 (69.5%)	130 (72.2%)
BLS * Ambulance	204 (23.2%)	99 (26.3%)	35 (19.4%)
ALS ** Ambulance	58 (6.6%)	16 (4.2%)	15 (8.3%)
Time of arrival, *n* (%)			
Morning (7 a.m.–3 p.m.)	297 (33.8%)	111 (29.4%)	60 (33.3%)
Evening (3 p.m.–11 p.m.)	276 (31.4%)	126 (33.4%)	60 (33.3%)
Night (11 p.m.–7 a.m.)	306 (34.8%)	140 (37.1%)	60 (33.3%)
Weekend, *n* (%)	260 (29.6%)	105 (27.9%)	41 (27.9%)
Season, *n* (%)			
Autumn	209 (23.8%)	74 (19.6%)	45 (25.0%)
Winter	233 (26.5%)	98 (26.0%)	45 (25.0%)
Spring	211 (24.0%)	103 (27.3%)	45 (25.0%)
Summer	226 (25.7%)	102 (27.1%)	45 (25.0%)
Assigned fall risk precautions, *n* (%)	254 (28.9%)	102 (27.1%)	45 (25.0%)
Comorbidities			
Cardiovascular, *n* (%)	222 (25.3%)	81 (21.5%)	62 (34.4%)
Neurologic, *n* (%)	75 (8.5%)	26 (6.9%)	21 (11.7%)
Respiratory, *n* (%)	67 (7.6%)	31 (8.2%)	21 (11.7%)
Malignancy, *n* (%)	69 (7.8%)	25 (6.6%)	11 (6.1%)
Outcome			
Hospital admission, *n* (%)	231 (26.3%)	99 (26.3%)	51 (28.3%)

* Basic life support (BLS); ** Advanced life support (ALS).

**Table 2:** Univariate analysis of predictors for hospital admission.

	Hospital Admission		
	No	Yes	*p*
	(*n* = 648	(*n* = 231)	
Demography			
Age (years), median (IQR)	45 (30–67)	65 (39–82)	<0.001
Female, *n* (%)	324 (50%)	110 (47.6%)	0.534
Urgency			
Triage levels, *n* (%)			<0.001
Immediate/emergency (1–2)	32 (4.9%)	28 (12.1%)	
Urgent (3)	255 (39.4%)	116 (50.2%)	
Semi- or non-urgent (4–5)	361 (55.7%)	87 (37.7%)	
Pulse (bpm), median (IQR)	80 (70–91)	83 (72–95)	0.038
Blood pressure (mmHg), median (IQR)			
Systolic	135 (123–151)	139.5 (125–156)	0.045
Diastolic	79 (69.5–88)	79 (68–88)	0.968
O_2_ Saturation (%), median (IQR)	99 (97–100)	98 (95–99)	<0.001
Temperature (°C), median (IQR)	36.8 (36.6–37)	36.8 (36.6–37)	0.973
Entry information			
Initial admitting diagnosis, *n* (%)			0.380
Medical	433 (66.8%)	147 (63.6%)	
Surgical/trauma	215 (33.2%)	84 (36.4%)	
Medical referral, *n* (%)	270 (41.7%)	101 (43.7%)	0.587
Mode of transportation, *n* (%)			<0.001
Private vehicle	485 (74.8%)	132 (57.1%)	
* BLS Ambulance	134 (20.7%)	70 (30.3%)	
** ALS Ambulance	29 (4.5%)	29 (12.6%)	
Time of arrival, *n* (%)			0.869
Morning (7:00 a.m.–3:00 p.m.)	222 (34.3%)	75 (32.5%)	
Evening (3:00 p.m.–11:00 p.m.)	201 (31.0%)	75 (32.5%)	
Night (11:00 p.m.–7:00 a.m.)	225 (34.7%)	81 (35.1%)	
Weekend, *n* (%)	174 (26.9%)	86 (37.2%)	0.003
Season, *n* (%)			0.039
Autumn	140 (21.6%)	69 (29.9%)	
Winter	171 (26.4%)	62 (26.8%)	
Spring	167 (25.8%)	44 (19.0%)	
Summer	170 (26.2%)	56 (24.2%)	
Assigned fall risk precautions, *n* (%)	143 (22.1%)	111 (48.1%)	<0.001
Comorbidities			
Cardiovascular, *n* (%)	106 (16.4%)	116 (50.2%)	<0.001
Neurologic, *n* (%)	31 (4.8%)	44 (19.0%)	<0.001
Respiratory, *n* (%)	41 (6.3%)	26 (11.3%)	0.015
Malignancy, *n* (%)	31 (4.8%)	38 (16.5%)	<0.001

* Basic life support (BLS); ** Advanced life support (ALS).

**Table 3:** Multivariable analysis of hospital admission.

	B	OR (95%CI)	*p*
Triage level: Immediate/Emergency/Urgent	0.369	1.447 (1.019–2.054)	0.039
O_2_ Saturation < 95%	1.199	3.316 (1.886–5.832)	<0.001
Assigned fall risk precautions	0.393	1.482 (0.995–2.206)	0.053
Comorbidities: Malignancy	0.592	1.807 (1.017–3.211)	0.044
Comorbidities: Cardiovascular	1.073	2.925 (1.974–4.335)	<0.001
Comorbidities: Neurologic	0.727	2.069 (1.159–3.693)	0.014
Weekend	0.453	1.573 (1.095–2.261)	0.014
Season: Autumn	0.594	1.812 (1.230–2.669)	0.003
Constant	−2.248		

Nagelkerke R Square = 0.247; Hosmer and Lemeshow Test, *p* = 0.572.

## Data Availability

The datasets generated and/or analyzed during the current study are available from the corresponding author on reasonable request.

## Abbreviations

The following abbreviations are used in this manuscript:

ED	Emergency department
MC-A	Medical center A (Tel Aviv Sourasky Medical Center)
MC-B	Medical center B (Shamir Medical Center)
AUC	Area under the (ROC) curve
ROC	Receiver operating characteristic
DS	Discrimination slope
IQR	Interquartile range
CTAS	Canadian Triage and Acuity Scale
BLS	Basic life support (ambulance)
ALS	Advanced life support (ambulance)
OR	Odds ratio
CI	Confidence interval
R^2^/pseudo R^2^	Pseudo Coefficient of Determination (Nagelkerke R^2^)
SPSS	Statistical Package for the Social Sciences
USA	United States of America

## Appendix A. The Conversion of Nomograms’ Total Points to Hospitalization Probability

**Total Points**	**Probability**
**4**	**0.1**
**13**	**0.11**
**21**	**0.12**
**29**	**0.13**
**36**	**0.14**
**43**	**0.15**
**49**	**0.16**
**55**	**0.17**
**61**	**0.18**
**67**	**0.19**
**72**	**0.2**
**77**	**0.21**
**82**	**0.22**
**87**	**0.23**
**91**	**0.24**
**96**	**0.25**
**100**	**0.26**
**105**	**0.27**
**109**	**0.28**
**113**	**0.29**
**117**	**0.3**
**121**	**0.31**
**125**	**0.32**
**128**	**0.33**
**132**	**0.34**
**136**	**0.35**
**140**	**0.36**
**143**	**0.37**
**147**	**0.38**
**150**	**0.39**
**154**	**0.4**
**157**	**0.41**
**161**	**0.42**
**164**	**0.43**
**167**	**0.44**
**171**	**0.45**
**174**	**0.46**
**178**	**0.47**
**181**	**0.48**
**184**	**0.49**
**188**	**0.5**
**191**	**0.51**
**194**	**0.52**
**198**	**0.53**
**201**	**0.54**
**204**	**0.55**
**208**	**0.56**
**211**	**0.57**
**214**	**0.58**
**218**	**0.59**
**221**	**0.6**
**225**	**0.61**
**228**	**0.62**
**232**	**0.63**
**236**	**0.64**
**239**	**0.65**
**243**	**0.66**
**247**	**0.67**
**250**	**0.68**
**254**	**0.69**
**258**	**0.7**
**262**	**0.71**
**266**	**0.72**
**271**	**0.73**
**275**	**0.74**
**279**	**0.75**
**284**	**0.76**
**288**	**0.77**
**293**	**0.78**
**298**	**0.79**
**303**	**0.8**
**309**	**0.81**
**314**	**0.82**
**320**	**0.83**
**326**	**0.84**
**332**	**0.85**
**339**	**0.86**
**346**	**0.87**
**354**	**0.88**
**362**	**0.89**
**371**	**0.9**
**381**	**0.91**
**391**	**0.92**
**403**	**0.93**
**417**	**0.94**
**433**	**0.95**
